# Tofersen decreases neurofilament levels supporting the pathogenesis of the SOD1 p.D91A variant in amyotrophic lateral sclerosis patients

**DOI:** 10.1038/s43856-024-00573-0

**Published:** 2024-07-25

**Authors:** Jochen H. Weishaupt, Péter Körtvélyessy, Peggy Schumann, Ivan Valkadinov, Ute Weyen, Jasper Hesebeck-Brinckmann, Kanchi Weishaupt, Matthias Endres, Peter M. Andersen, Martin Regensburger, Marie Dreger, Jan C. Koch, Julian Conrad, Thomas Meyer

**Affiliations:** 1 Division of Neurodegenerative Disorders, Neurological University Clinic Mannheim, Medical Faculty Mannheim, Mannheim Center for Translational Neurosciences, Heidelberg University, Mannheim, Germany; 2 Charité – Universitätsmedizin Berlin, Humboldt-Universität zu Berlin, Center for ALS and other Motor Neuron Disorders, Berlin, Germany; 3 German Center for Neurodegenerative Diseases (DZNE), Research Site Magdeburg, Magdeburg, Germany; 4 Ambulanzpartner Soziotechnologie APST GmbH, Berlin, Germany; 5 Berufsgenossenschaftliches Universitätsklinikum Bergmannsheil, Department of Neurology, Center for ALS and other Motor Neuron Disorders, Bochum, Germany; 6 Department of Clinical Sciences, Neuroscience, Umeå University, Umeå, Sweden; 7 Department of Molecular Neurology, Friedrich-Alexander-Universität Erlangen-Nürnberg, Erlangen, Germany; 8 Deutsches Zentrum Immuntherapie (DZI), University Hospital Erlangen, Erlangen, Germany; 9 Clinic for Neurology, University Medicine Göttingen, Göttingen, Germany

**Keywords:** Neurological disorders, Predictive markers

## Abstract

**Background:**

Since the antisense oligonucleotide tofersen has recently become available for the treatment of amyotrophic lateral sclerosis (ALS) caused by mutations in *SOD1*, determining the causality of the over 230 *SOD1* variants has become even more important. The most common *SOD1* variant worldwide is p.D91A (c.272A > C), whose causality for ALS is contested when in a heterozygous state. The reason is the high allele frequency of *SOD1*^*D91A*^ in Europe, exceeding 1% in Finno-Scandinavia.

**Methods:**

We present the clinical disease course and serum neurofilament light chain (NfL) results of treating 11 patients either homo- or heterozygous for the *SOD1*^*D91A*^ allele for up to 16 months with tofersen.

**Results:**

Tofersen decreases serum neurofilament levels (sNFL), which are associated with the ALS progression rate, in the 6 ALS patients homozygous for *SOD1*^*D91A*^. We observe significantly lower sNfL levels in the 5 patients heterozygous for *SOD1*^*D91A*^. The results indicate that both mono- and bi-allelic *SOD1*^*D91A*^ are causally relevant targets, with a possibly reduced effect size of *SOD1*^*D91Ahet*^.

**Conclusions:**

The finding is relevant for decision making regarding tofersen treatment, patient counseling and inclusion of *SOD1*^*D91A*^ patients in drug trials. As far as we are aware, the approach is conceptually new since it provides evidence for the causality of an ALS variant based on a biomarker response to gene-specific treatment.

## Plain language summary

Amyotrophic lateral sclerosis (ALS) is a disease that can be inherited which affects nerve cells in the brain and spinal cord. Changes within a gene called *SOD1* that result in a mutation named p.D91A can lead to the development of ALS. People have two copies of the *SOD1* gene. It has been unclear whether the presence of only one copy of p.D91A can cause ALS. We treated ALS patients with the p.D91A variant of *SOD1* with a drug called tofersen. We found that a marker of disease progression was reduced in patients with one or two copies of the p.D91A mutation. This suggests that the presence of just one p.D91A variant of *SOD1* contributes to disease development. This information could be used to improve treatment decisions for people with ALS.

## Abstract (web summary)

Weishaupt, Körtvélyessy, et al. investigate the role of a genetic variant in amyotrophic lateral sclerosis (ALS) causation. Reduction of serum neurofilament light chain levels upon treatment with the SOD1-specific drug tofersen indicates engagement of a relevant target and thus causality of the variant p.D91A in SOD1.

## Introduction

Amyotrophic lateral sclerosis (ALS) is an adult-onset neurodegenerative disease affecting predominantly the cortical and spinal motoneurons. Some 5-10% of patients report a positive family history for the disease (familial ALS; fALS)^1^. Heterozygous mutations in the gene encoding superoxide dismutase 1 (*SOD1)* are detected in 9-23% of fALS and 2% of sporadic ALS (sALS) patients^2,3^. *SOD1* mutations act by a toxic gain-of-function^3^. The intrathecally administered antisense-oligonucleotid tofersen binds to *SOD1* mRNA and reduces SOD1 protein expression^4^. It represents the first gene-specific therapy for ALS and has recently been approved or is available within early access programs in several countries.

While more than 230 different ALS-associated *SOD1* mutations have been identified to date, p.D91A (*SOD1*^*D91A*^; c.272A>C) takes a unique position distinct from other *SOD1* mutations: ALS caused by *SOD1*^*D91A*^ is most frequently inherited as a recessive trait, while all other mutations in *SOD1* are dominantly inherited^5^. ALS caused by the homozygous but not heterozygous *SOD1*^D91A^ shows a distinct clinical phenotype including an unusual slow progression^5,6^. The most notable aspect of the p.D91A mutation in *SOD1* is its high frequency. While the vast majority of *SOD1* mutations are rare or absent in control cohorts, the allele frequency of heterozygous *SOD1*^*D91A*^ is in the order of 0.1% in non-Finnish Europeans and exceeds 1% in Northern Sweden and Finland (https://gnomad.broadinstitute.org/)^5^. Moreover, in contrast to other *SOD1* mutations, *SOD1* protein deposits were not detectable in an autopsy case of a patient with a heterozygous *SOD1*^*D91A*^ mutation^7^. For these reasons, the causality of the *SOD1*^*D91A*^ variant, especially when in a heterozygous state, has been contested as a cause of ALS since its discovery in 1993. On the other hand, *SOD1*^*D91Ahet*^ has been found in a substantial number of ALS patients also in populations where the allele frequency is very low^8^ and contributes to the *SOD1*-associated signal in ALS GWAS studies^9^, providing arguments for a causal contribution of this variant.

Only two patients with a homozygous and none with a heterozygous *SOD1*^*D91A*^ variant were recruited into the tofersen phase III study VALOR^4^. Considering the high population frequency of heterozygous *SOD1*^*D91A*^, the debate surrounding its causality and the recent availability of a *SOD1*-specific drug, there is an urgent need to settle whether ALS patients with a *SOD1*^*D91A*^ mutation should be treated with tofersen.

Neurofilament light chain (NfL) protein levels are elevated in ALS patients^10,11^ and correlate with prognosis^12,13^. The pivotal tofersen VALOR study^4^ and also first real-world data^14,15^ revealed a robust decrease of serum neurofilament light chain (sNfL) concentrations upon tofersen treatment in patients with *SOD1* mutations of determined pathogenicity. The US Federal Drug Administration (FDA) approved sNfL levels as a surrogate marker for engagement of a relevant therapeutic target (*SOD1*) in *SOD1*-ALS.We hypothesized that a biomarker response to the *SOD1*-directed ASO tofersen could prove the causality of this mutation in both hetero- and homozygous *SOD1*^*D91A*^ mutant patients.

Thus, we assess the sNfL levels and clinical course of 11 patients with mono- or bi-allelic *SOD1*^*D91A*^ mutation. We find that tofersen treatment of these patients leads to a reduction of sNfL, corroborating the causality of both homo- and heterozygous p.D91A mutations.

## Methods

### Patient recruitment

Data analysis was performed in patients with ALS and a *SOD1*^*D91A*^ mutation who were treated with tofersen for at least 4 months.

Tofersen treatment was provided within a compassionate use program/early access program (see ethics statement below). To be eligible to participate in this early access/compassionate use program, participants were required to meet all of the following eligibility criteria: Participants could not be included in an ongoing clinical trial and could not be satisfactorily treated with a medicinal product authorized for sale within the territorial scope of the Medicinal Product Act. A written signed informed consent needed to have been obtained and confirmed. Participants had to have the ability to understand the purpose and risks of the program and have provided signed and dated informed consent and authorization to use protected health information (PHI) in accordance with national and local participant privacy regulations. In the case that a participant was legally incapable of providing informed consent, the participant’s legally authorized representative had to provide the informed consent. Participants providing their own informed consent had to be aged ≥18 years at the time they provided informed consent. Participants had to have weakness attributable to ALS and associated with a mutation in the superoxide dismutase 1 (SOD1) gene (SOD1-ALS). Participants had to be medically able to undergo the program procedures, as determined by the Treating HCP. Participants of childbearing potential had to agree to practice effective contraception during the program and be willing and able to continue such contraception methods for 5 months after their last treatment dose in the CUP.

The following patients were excluded from inclusion if they had: any comorbidities or conditions that, in the opinion of the treating health care provider (HCP), would unacceptably increase the risk of participation, including contraindications to lumbar punctures (LPs); anticipated need, in the opinion of the Treating HCP, for the administration of any antiplatelet or anticoagulant medication that could not be safely held before and/or after an LP procedure according to local or institutional guidelines and/or treating HCP determination; previous or current participation in a clinical trial of tofersen; use of an investigational medicinal product (IMP) for ALS within 5 half-lives of the IMP before the first dose of tofersen; or had a primary place of residency outside the country of treatment.

Patients were identified at five multidisciplinary German ALS centers in Berlin (6 patients), Mannheim (2 patients), Bochum, Erlangen and Göttingen (1 patient each). SOD1 mutation status was assessed by clinical diagnostic testing. ALSFRS-R data were assessed by self-rating either on a printed form or using the ALS-App15. Data were collected between March 2022 and July 2023. The ALS progression rate (ALS-PR) was calculated using the following formula: (48 - ALSFRS-R divided by disease duration in months).

Slow vital capacity (SVC) was measured in percent of the predicted value (corrected for height, age, sex, and weight).

### Ethics statement

Patients were treated within an early access program (EAP) for tofersen approved at the EU level by the EMA and conducted in several European countries. Treatment was through the drug hardship program (compassionate use program) in accordance with § 21, section Methods No. 6 SGB V in conjunction with article 83 of the Regulation (EC) No 726/2004 of the European Parliament and of the Council of 31 March 2004. This was implemented in Germany in accordance with the marketing regulation of medicines without approval or without approval in hardship cases (drug hardship cases regulation—AMHV). IRB board and ethics committees (ethics committee II of the Heidelberg University, Mannheim; ethics committee of the Charitee, Berlin; ethics committee of the Friedrich-Alexander-Universität Erlangen-Nürnberg, Erlangen; ethics committee of the medical faculty RUB, Bochum; ethics committee of the medical faculty Göttingen, Göttingen) confirmed that they were not responsible for approving the treatment for compassionate use and required approval from the EU as an EAP. Tofersen was made available through the Biogen early access program via ClinigenDirect (clinigengroup.com) for patients with diagnosed ALS and a mutation in *SOD1*. Participants gave written informed consent according to CARE guidelines and in compliance with the Declaration of Helsinki principles.

### Serum NfL measurement

Serum NfL (sNfL) concentrations were analyzed at the ALS center in Berlin using single-molecule analysis technology (SIMOA) and the commercially available NfL Advantage kit (Quanterix, Inc, Billerica, Massachusetts, USA).

### Tofersen treatment

Tofersen was made available through the early access program via ClinigenDirect (clinigengroup.com). Intrathecal treatment was performed according to the protocol of the VALOR study^4^.

### Statistics and reproducibility

Descriptive statistics were used (frequency in percent, mean, and ranges). We used Graph Pad Prism 8.1 for statistical analyses. The Shapiro-Wilk test revealed that both cohorts are distributed normally. In order to calculate the best fit obeying the order of measurements within one individual patient linear regressions per patient are used to assess whether the decline of sNfL levels is significant. We also used the repeated measures ANOVA with Greenhouse-correction together with the test for linear trend to check for significant differences within each timepoint of sNfL within one patient, since other statistical test do not compare levels individually in longitudinal series but group-wise. For the comparison of the sNfL levels between both cohorts a non-paired Wilcoxon signed rank test was used. sNfL levels were measured in duplicates (i.e. technical replicates). Since this work describes a case series, biological replicates were not possible.

### Reporting summary

Further information on research design is available in the Nature Portfolio Reporting Summary linked to this article.

## Results

### Patient recruitment and characteristics

We recruited five ALS patients heterozygous and six ALS patients homozygous for the c.272A>C/p.D91A mutation in *SOD1* from five specialized German ALS outpatients clinics. Patient characteristics are detailed in Suppl. Table 1. We included all patients into the German tofersen early access program and treated the patients according to the VALOR study^4^. Side effects were related to the lumbar puncture, such as headache and back pain. Symptoms suggesting radiculitis, increased intracranial pressure or myelitis were absent. The treatment period was 4 to 16 months at the time of this report, and the total number of tofersen injections was 11.09 (between 5–17 injections) per patient. At baseline, there were no significant differences in mean ALSFRS-R values (*p* = 0.95), disease duration (*p* = 0.84) or slow vital capacity (SVC; *p* = 0.42) between *SOD1*^*D91Ahet*^ and *SOD1*^*D91Ahom*^ patients (Supplementary Table 1). We measured significantly higher sNfL values in the group of *SOD1*^*D91Ahom*^ patients when compared to *SOD1*^*D91Ahet*^ (*p* = 0.03; Suppl. Table 1; Supplementary data 1). 9 patients were treated with riluzole before and during tofersen treatment, while 1 patient tapered riluzole during tofersen treatment.

### Clinical disease progression

Most patients displayed a relatively slow disease progression. The ALSFRS-R progression rate (ALS-PR; mean decay in ALSFRS-R points per month) was 0.23 ± 0.1 for the homozygous p.D91A patients and 0.26 ± 0.04 (mean ± S.D.) in the heterozygous p.D91A group at initiation of tofersen treatment. Considering the small patient cohort, diverse clinical phenotype and different observation periods for fast and slowly progressing patients, a statement regarding a mean change in ALS-PR or slow vital capacity (SVC; as measured in percent of the predicted value (corrected for height, age, sex, and weight)) under tofersen treatment could not be made (Supplementary Fig. 1a–d). A tendency to slower disease progression or improved SVC was noted in some *SOD1*^*D91Ahet*^ and *SOD1*^*D91Ahom*^ patients (Suppl. Fig. 1). SVC ranged from −8% to 32% at the last measurement of each individual when compared to the basal value. All homozygous *SOD1*^*D91A*^ patients reported subjective improvement of symptoms relevant for their activities of daily living.

### Serum neurofilament light chain levels

**Fig. 1:**
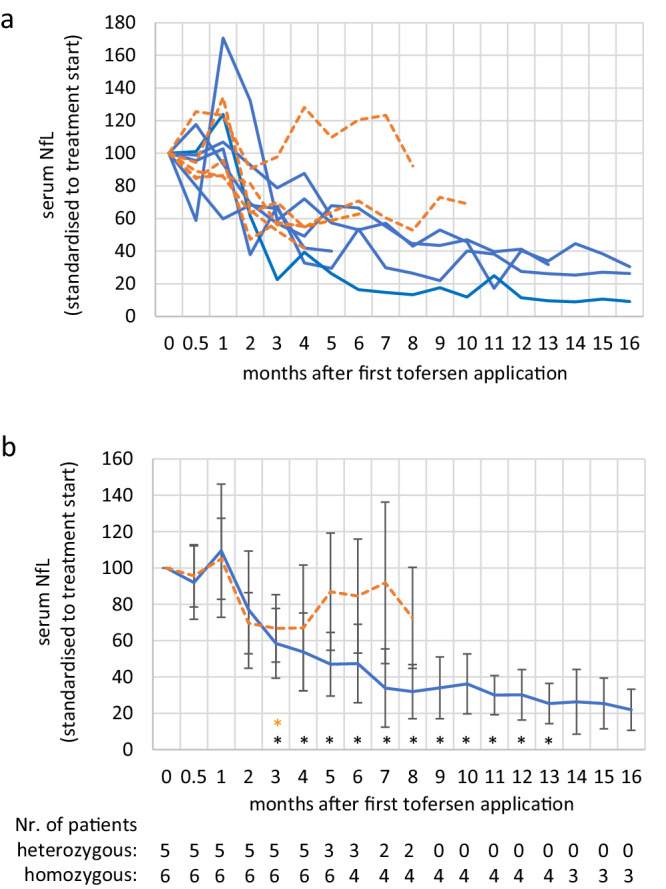
Serum NfL course in tofersen-treated *SOD1*^*D91A*^ mutant patients. **a** Serum neurofilament light chain (sNfL) levels during treatment with tofersen. For better comparability, values were normalized to the timepoint directly before the start of treatment. Values from patients with heterozygous mutations are depicted with a dashed orange line, homozygous patients are depicted in blue. **b** Mean sNfL values ± S.D. of patients shown in b). Values were normalized to the timepoint directly before the start of treatment. Asterisk indicates significant reduction when compared to start of treatment in patients with a homozygous mutation (repeated measures ANOVA*: *p* < 0.05; orange asterisk (top row of asterisks): refers to heterozygous patients; black asterisks (bottom row of asterisks): homozygous patients; the exact p values can be found in Supplementary Data 1). Two NfL measurements have been missed for a heterozygous and homozygous patient at months 5 and 7, respectively. After the sixth injection the number of heterozygous patients was too low for a group-based analysis. The statistics are based on absolute values not normalized to treatment start.

Serum neurofilament light chain levels (sNfL) correlate with the speed of disease progression in ALS^12,13^. We observed that all homozygous *SOD1*^*D91A*^ patients displayed a significant (repeated measures ANOVA, *p* < 0.05, F(2.1011;8.766) = 4.793) reduction in sNfL concentration starting after the fourth intrathecal application (i.e. measured in serum collected immediately before the fifth application; Fig. 1a, b; Supplementary data 1). In one patient, sNfL values were found to be stable below 10% of the concentration measured at the beginning of treatment after 10 months of treatment (Fig. 1a). We also found a significant reduction of mean sNfL concentrations in heterozygously mutant *SOD1*^*D91A*^ patients (Fig. 1a, b) after the fourth injection (repeated measures ANOVA, *p* < 0.05, F(2.191;8.766) = 5.015) but not after the fifth (*p* > 0.05). The number of heterozygous patients was too low for applying the repeated measures ANVOA after the sixth injection.

Therefore, we additionally assessed the individual sNfL trajectories and performed individual linear regression analysis for every patient. Three out of five heterozygous p.D91A patients showed a significant reduction in sNfL after the fifth treatment, one a trend (*p* = 0.08) and one did not respond with a decrease in sNfL (Suppl. Fig. 2a). Five out of six homozygous patients had a significant reduction in sNfL after 6 injections (Supplementary Fig. 2b).

## Discussion

We employed sNfL as a biomarker for tofersen target engagement and thus a causal role of the *SOD1*^*D91A*^ mutation in 11 patients with this mutation in heterozygous or homozygous state.

The short observation period, slow disease progression and small cohort size make a robust statement on clinical treatment response in this study difficult. However, several studies have shown that neurofilaments remain stable during ALS and are associated with disease aggressiveness^11,13^. We observed a significant decrease of sNfL values in the serum of both *SOD1*^*D91Ahom*^ and *SOD1*^*D91Ahet*^ patients during tofersen treatment. In one patient, sNfL fell to consistently less than 10%. This result strongly suggests that the *SOD1* antisense-oligonucleotide tofersen is leading to a positive response of a biomarker for disease aggressiveness in patients with a *SOD1*^*D91A*^ mutation, confirming its causal contribution in a monogenic sense (homozygous variant) and suggesting at least a role as a risk factor contributing to ALS pathogenesis (heterozygous state). However, although less likely, we cannot exclude and have to consider alternative explanations. For example, since SOD1 is a highly expressed protein, lowering SOD1 protein levels could be sufficient to relieve the proteostatic burden in neurons and lead to a reduction in neurofilament levels. Moreover, tofersen or the lowering of SOD1 protein in general might alter the processing of neurofilaments and directly result in a decrease of neurofilaments.

Four out of five *SOD1*^*D91Ahet*^ patients displayed a progressive decline in sNfL levels with tofersen treatment. This represents evidence for a causal contribution also of the heterozygous *SOD1*^*D91A*^ mutation, albeit with a substantially smaller effect size when compared to the homozygous state. This attenuated sNfL response to tofersen treatment is consistent with *SOD1*^*D91A*^ considered as a variant biochemically intermediate between wild-type *SOD1* and more penetrant mutations^5^. However, *SOD1*^*D91Ahet*^ patients usually present with a more variable and often times more severe phenotype than patients with *SOD1*^*D91Ahom* 6^. *SOD1*^*D91Ahet*^ may thus represent a weaker genetic variant in the context of multifactor causation, rather than a Mendelian mutation. It could be considered a risk factor for ALS, meaning that interaction with one or more unknown additional factors (e.g. TDP-43^7^) may be necessary to cause ALS. This hypothesis is in line with the diverse phenotype^5,6^ and tofersen treatment response of *SOD1*^*D91Ahet*^ patients.

The findings show that both homozygous and heterozygous *SOD1*^*D91A*^ mutations are targets for treatment with tofersen. This is important for decision-making regarding tofersen treatment, patient counseling, and inclusion of *SOD1*^*D91A*^ patients in drug trials. In a broader view, it suggests that gene-targeted treatment might not only be reserved for fully penetrant genetic ALS variants but could also be effective for risk variants. Patients with a heterozygous mutation should be counseled that the treatment effect may be smaller than in the case of a homozygous mutation, although this statement awaits confirmation. The currently small sample size and comparably short follow-up will require further follow-up of more tofersen-treated patients with p.D91A mutation in *SOD1*. Our approach provides evidence for the previously unclear causality of a neurodegeneration-related genetic variant based on biomarker response to a gene-specific treatment.

*Note added in proof:* The European Commission has meanwhile approved Qalsody (tofersen) for the treatment of ALS associated with mutations in the *SOD1* gene.

### Supplementary information

Supplementary Information

Description of Additional Supplementary Files

Supplementary data 1

Reporting Summary

## Data Availability

The numerical (source) data underlying Fig. 1 can be found in Supplementary Data 1.
