# Supplementary material for: Tofersen decreases neurofilament levels supporting the pathogenesis of the SOD1 p.D91A variant in amyotrophic lateral sclerosis patients
Source: Commun Med (Lond). 2024 Jul 25;4:150. doi: 10.1038/s43856-024-00573-0 (PMC11272917; doi:10.1038/s43856-024-00573-0)
Supplement: Supplementary file 1 — Supplementary Information [file 43856_2024_573_MOESM1_ESM.pdf]

Suppl. Table 1. Basal patient characteristics.

Patient Nr.	Zygosity of SOD1 p.D91A mutation	Sex (ratio f:m)	Age at onset (years); individual values removed for anonymi-sation	Site of onset	UMN or LMN predominance	Disease duration at start of tofersen treatment (months)	ALSFRS-R at start of tofersen treatment	ALS-PR at start of tofersen treatment	sNfL concentration at start of tofersen treatment (pg/ml)	Total number of tofersen injections
1	heterozygous	f		arm	UMN/LMN	19	42	0,32	57,84	10
2	heterozygous	f		leg	LMN	45	37	0,24	49,97	8
3	heterozygous	f		leg	UMN/LMN	53	28	0,38	36,66	10
4	heterozygous	f		leg	UMN/LMN	70	32	0,23	16,81	6
5	heterozygous	m		leg	UMN/LMN	44	42	0,12	37,2	5
Mean of patients with heterozygous mutation	heterozygous	4:1	55,8			46,2	36,2	0,26	39,70	7,80
6	homozygous	f		leg	UMN/LMN	42	45	0,07	61,59	18
7	homozygous	m		leg	UMN	32	46	0,06	63,26	18
8	homozygous	f		leg	UMN/LMN	22	42	0,27	107,01	18
9	homozygous	f		leg	UMN/LMN	105	25	0,22	55,7	7
10	homozygous	m		leg	UMN/LMN	40	21	0,68	60,4	7
11	homozygous	m		leg	UMN/LMN	16	n.a.	0,07	39,06	15
Mean of patients with homozygous mutation	homozygous	3:3	53,2			42,8	35,8	0,23	64,50	13,83
Mean of all patients	Combined heterozygous and homozygous mutation	7:4	54,4			44,4	36,0	0,24	53,23	11,09

n.a. = not available; ALS-PR: ALS progression rate: 48 - ALSFRS-R divided by disease duration [months]; UMN/LMN predominance: dominance of upper or lower motoneuron symptoms, respectively; NfL: Neurofilament light chain.

Suppl. Fig. 1

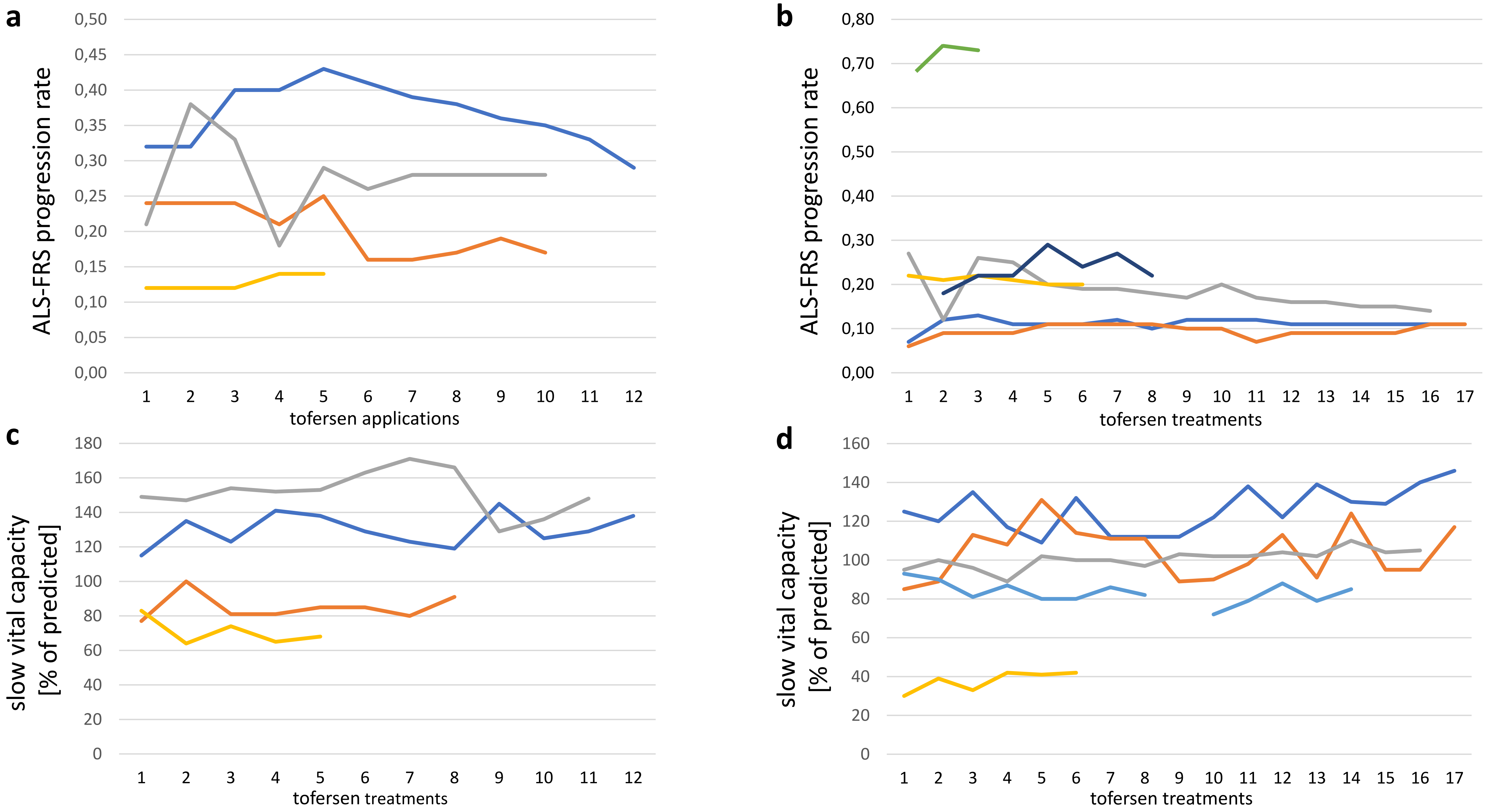
a
0,50
0,45
0,40
0,35
0,30
0,25
0,20
0,15
0,10
0,05
0,00
ALS-FRS progression rate
1 2 3 4 5 6 7 8 9 10 11 12
tofersen applications
b
0,80
0,70
0,60
0,50
0,40
0,30
0,20
0,10
0,00
ALS-FRS progression rate
1 2 3 4 5 6 7 8 9 10 11 12 13 14 15 16 17
tofersen treatments
c
180
160
140
120
100
80
60
40
20
0
slow vital capacity [% of predicted]
1 2 3 4 5 6 7 8 9 10 11 12
tofersen treatments
d
160
140
120
100
80
60
40
20
0
slow vital capacity [% of predicted]
1 2 3 4 5 6 7 8 9 10 11 12 13 14 15 16 17
tofersen treatments

Legend for Suppl. Fig. 1: ALSFRS-R progression rate and course of slow vital capacities (SVC). Course of the mean ALS progression rate (ALS-PR; decline in ALSFRS-R points per month) in individual patients with heterozygous (**a**) or homozygous (**b**) p.D91A mutation in *SOD1* during treatment with tofersen. Longitudinal course of the slow vital capacity (SVC; measured in percent of the predicted value (corrected for height, age, sex, and weight)) in patients with heterozygous (**c**) or homozygous (**d**) p.D91A mutation in *SOD1* during treatment with tofersen. Data are given if at least two timepoints were available, we did not have the data for one and two patients for ALS-PR and SVC, respectively.

Suppl. Fig. 2

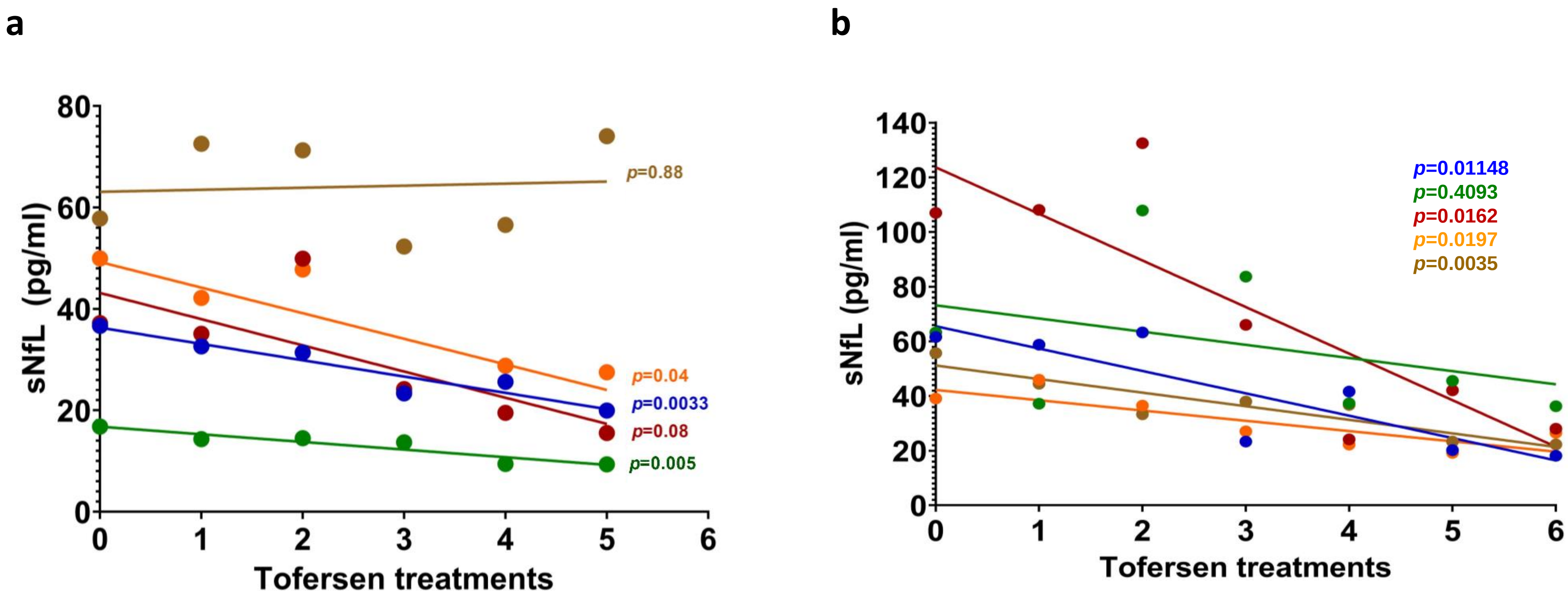
a
80
60
40
20
0
sNfL (pg/ml)
0
1
2
3
4
5
6
Tofersen treatments
p=0.88
p=0.04
p=0.0033
p=0.08
p=0.005
b
140
120
100
80
60
40
20
0
sNfL (pg/ml)
0
1
2
3
4
5
6
Tofersen treatments
p=0.01148
p=0.4093
p=0.0162
p=0.0197
p=0.0035

Legend for Suppl. Fig. 2: Simple linear regression analysis of individual patient sNfL levels. Results for heterozygous (**a**) and homozygous (**b**) *SOD1*^{D91A} patients are displayed separately, and p values of the linear regression analysis are displayed for each individual patient in paired colours. Analysis was based on timepoints when all patients in the respective group received tofersen, and excluded later timepoints when first patients dropped out. Note that not every data was available at each timepoint. Numbers on the x-axis mean number of tofersen injections.
