# Supplementary material for: Tofersen decreases neurofilament levels supporting the pathogenesis of the SOD1 p.D91A variant in amyotrophic lateral sclerosis patients
Source: Commun Med (Lond). 2024 Jul 25;4:150. doi: 10.1038/s43856-024-00573-0 (PMC11272917; doi:10.1038/s43856-024-00573-0)
Supplement: Supplementary file 2 — Description of Additional Supplementary Files [file 43856_2024_573_MOESM2_ESM.pdf]

Description of Additional Supplementary Files

File name- Supplementary data

File description- Numerical data and exact p values for figure 1.
