# Supplementary material for: Tofersen decreases neurofilament levels supporting the pathogenesis of the SOD1 p.D91A variant in amyotrophic lateral sclerosis patients
Source: Commun Med (Lond). 2024 Jul 25;4:150. doi: 10.1038/s43856-024-00573-0 (PMC11272917; doi:10.1038/s43856-024-00573-0)
Supplement: Supplementary file 4 — Reporting Summary [file 43856_2024_573_MOESM4_ESM.pdf]

Corresponding author(s): Jochen Weishaupt
Thomas Meyer

Last updated by author(s): 2024-6-10

Reporting Summary

Nature Portfolio wishes to improve the reproducibility of the work that we publish. This form provides structure for consistency and transparency in reporting. For further information on Nature Portfolio policies, see our Editorial Policies and the Editorial Policy Checklist.

Statistics

For all statistical analyses, confirm that the following items are present in the figure legend, table legend, main text, or Methods section.

n/a	Confirmed	
☐	☒	The exact sample size (*n*) for each experimental group/condition, given as a discrete number and unit of measurement
☐	☒	A statement on whether measurements were taken from distinct samples or whether the same sample was measured repeatedly
☐	☒	The statistical test(s) used AND whether they are one- or two-sided<br>*Only common tests should be described solely by name; describe more complex techniques in the Methods section.*
☐	☒	A description of all covariates tested
☐	☒	A description of any assumptions or corrections, such as tests of normality and adjustment for multiple comparisons
☐	☒	A full description of the statistical parameters including central tendency (e.g. means) or other basic estimates (e.g. regression coefficient) AND variation (e.g. standard deviation) or associated estimates of uncertainty (e.g. confidence intervals)
☐	☒	For null hypothesis testing, the test statistic (e.g. *F*, *t*, *r*) with confidence intervals, effect sizes, degrees of freedom and *P* value noted<br>*Give P values as exact values whenever suitable.*
☐	☒	For Bayesian analysis, information on the choice of priors and Markov chain Monte Carlo settings
☐	☒	For hierarchical and complex designs, identification of the appropriate level for tests and full reporting of outcomes
☐	☒	Estimates of effect sizes (e.g. Cohen's *d*, Pearson's *r*), indicating how they were calculated

Our web collection on statistics for biologists contains articles on many of the points above.

Software and code

Policy information about availability of computer code

Data collection	Serum Neurofilament light chain levels were measured and transfered to a data matrix.
Data analysis	Data Analysis was in a first step done with Excel, and then with StatPlus Mac as well as Graph Pad Prism.

For manuscripts utilizing custom algorithms or software that are central to the research but not yet described in published literature, software must be made available to editors and reviewers. We strongly encourage code deposition in a community repository (e.g. GitHub). See the Nature Portfolio guidelines for submitting code & software for further information.

Data

Policy information about availability of data

All manuscripts must include a data availability statement. This statement should provide the following information, where applicable:

- Accession codes, unique identifiers, or web links for publicly available datasets
- A description of any restrictions on data availability
- For clinical datasets or third party data, please ensure that the statement adheres to our policy

There are no additional publicly available datasets other than provided in the manuscript, without restriction on data availability.

Research involving human participants, their data, or biological material

Policy information about studies with human participants or human data. See also policy information about sex, gender (identity/presentation), and sexual orientation and race, ethnicity and racism.

Reporting on sex and gender	In this clinical case series both male and female patients have been included. The exact numbers and ratios are clearly outlined in the manuscript, but were not under our control, since the data are not derived from a clinical trial.
Reporting on race, ethnicity, or other socially relevant groupings	Our manuscript does not contain any social or ethnical categorization or respective analysis. Patients were recruited according to their SOD1 mutation status and tofersen treatment only.
Population characteristics	Patients were recruited and analysed according to their SOD1 mutation status and eligibility for the tofersen early access program solely. All patients are of central European origin.
Recruitment	Patients were recruited according to their SOD1 mutation status and eligibility for the tofersen early access program. Patitents were recruited in five German specialized ALS outpatients clinics.
Ethics oversight	Patients were treated within an early access program for tofersen approved by the EMA and conducted in several European countries. The legal framework is the drug hardship program (compassionate use program) in accordance with § 21, section 2, No. 6 SGB V in conjunction with article 83 of the Regulation (EC) No 726/2004 of the European Parliament and of the Council of 31 March 2004 and in conjunction with the marketing regulation of medicines without approval or without approval in hardship cases (drug hardship cases regulation – AMHV) in Germany. IRB board and ethics committees (ethics committee II of the Heidelberg University, Mannheim; ethics committee of the Charitee, Berlin; ethics committee of the Friedrich-Alexander-Universität Erlangen-Nürnberg, Erlangen; ethics committee of the medical faculty RUB, Bochum; ethics committee of the medical faculty Göttingen, Göttingen) were informally asked for advice but declared not to be responsible for compassionate use treatments, and that this is not a clinical study. Tofersen was made available through the Biogen early access program via ClinigenDirect (clinigengroup.com) for patients with diagnosed ALS and a mutation in SOD1. Participants gave written informed consent according to CARE guidelines and in compliance with the Declaration of Helsinki principles.Tofersen was made available through the Biogen early access program via ClinigenDirect (clinigengroup.com). Participants gave written informed consent, according to CARE guidelines and in compliance with the Declaration of Helsinki principles.

Note that full information on the approval of the study protocol must also be provided in the manuscript.

Field-specific reporting

Please select the one below that is the best fit for your research. If you are not sure, read the appropriate sections before making your selection.

☒ Life sciences ☐ Behavioural & social sciences ☐ Ecological, evolutionary & environmental sciences

For a reference copy of the document with all sections, see nature.com/documents/nr-reporting-summary-flat.pdf

Life sciences study design

All studies must disclose on these points even when the disclosure is negative.

Sample size	Case series including 11 patients.
Data exclusions	No data have been excluded.
Replication	Since this is a clinical case series of patients in a compassionate use program there was no replication.
Randomization	Since this is a clinical case series the patients were not randomized (no clinical trial was performed).
Blinding	Since this is a clinical case series of patients in a compassionate use program and the patients were all tretated with tofersen, there was no blinding.

Reporting for specific materials, systems and methods

We require information from authors about some types of materials, experimental systems and methods used in many studies. Here, indicate whether each material, system or method listed is relevant to your study. If you are not sure if a list item applies to your research, read the appropriate section before selecting a response.

Materials & experimental systems

n/a	Involved in the study
☒	☐ Antibodies
☒	☐ Eukaryotic cell lines
☒	☐ Palaeontology and archaeology
☒	☐ Animals and other organisms
☐	☒ Clinical data
☒	☐ Dual use research of concern
☒	☐ Plants

Methods

n/a	Involved in the study
☒	☐ ChIP-seq
☒	☐ Flow cytometry
☒	☐ MRI-based neuroimaging

Clinical data

Policy information about clinical studies

All manuscripts should comply with the ICMJE guidelines for publication of clinical research and a completed CONSORT checklist must be included with all submissions.

Clinical trial registration	The clinical data (data obtained in a clinical setting) are not derived from from a clinical trial, therefore there is no clinical trial registration number.
Study protocol	The clinical data (data obtained in a clinical setting) are not derived from from a clinical trial, therefore there is no predefined clinical trial protocol.
Data collection	Data were collected from patients that were treated within an earyl access program for tofersen. Patients were identified at five multidisciplinary German ALS centers in Berlin (6 patients), Mannheim (2 patients), Bochum, Erlangen and Göttingen (1 patient each). Data were collected between March 2022 and July 2023.
Outcomes	Since this is a clinical case series of patients in a compassionate use program there were no pre-defined outcome measures.

Plants

Seed stocks	No plants of any kind are examined or used in this clinical case series.
Novel plant genotypes	No plants of any kind are examined or used in this clinical case series.
Authentication	No plants of any kind are examined or used in this clinical case series.
